# Supplementary material for: Are carbon emissions trading and green financial instruments synergistic? -Comprehensive quantitative research based on content analysis
Source: PLoS One. 2024 Mar 7;19(3):e0298601. doi: 10.1371/journal.pone.0298601 (PMC10919601; doi:10.1371/journal.pone.0298601)
Supplement: S1 Data — (ZIP) [file pone.0298601.s001.zip › Data/地级市Z_F工作报告.zip!/╕ú╜¿/╚¬╓▌╩╨/└┤╫╘╣1⁄2╓┌║┼í╛┬φ┐╦╩2╛▌═°í┐_2019.docx]

各位代表：

　　现在，我代表泉州市人民政府向大会报告工作，请予审议，并请各位政协委员和其他列席人员提出意见。

　　一、2018年政府工作回顾

　　过去一年，在中共泉州市委领导下，市人民政府深入学习贯彻习近平新时代中国特色社会主义思想和党的十九大精神，全面落实中央、省和市委决策部署，认真执行市人大及其常委会决议决定，坚持稳中求进，落实新发展理念，传承弘扬“晋江经验”，深化供给侧结构性改革，制定实施赶超三年行动计划，打好三大攻坚战，做好“六稳”工作，全力推进产业升级、城乡统筹、民生改善。全市实现生产总值8458亿元、增长8.9%（预计，下同），规模以上工业增加值3860亿元、增长9.1%，一般公共预算总收入861.1亿元、增长6.5%，一般公共预算收入474.2亿元、增长7.2%，居民人均可支配收入36080元、增长8.5%，完成市十六届人大二次会议确定的主要目标任务，高质量赶超迈出坚实一步。一年来主要工作和成效是：

　　（一）全力稳增长，经济运行稳中向好。战役式推进“项目攻坚2018”。分主题连续开展“大干40天、比拼开门红”、审批服务月、招商突破行动、年底的“百日大会战”等活动。列入省“五个一批”项目3580个、总投资3.2万亿元，居全省第一；新签约产业项目356个、总投资3662亿元。423个在建重点项目完成投资1579亿元。全市固定资产投资增长13.1%。精准帮扶企业。出台稳定外资外贸、稳定工业、奖励工业用电等扶持政策，深入开展“领导干部一线办公”活动周，建立企业诉求“周收集、旬解决、月反馈”制度，兑现企业扶持资金47亿元，落实税收减免115亿元，“清费减负”40亿元。稳妥应对中美经贸摩擦影响，精心举办泉州商品博览会等系列展销活动，全年出口完成1180亿元、增长13%。坚决打好防范化解重大风险攻坚战。健全政府债务风险防控机制，加强增量债务控制和存量债务化解。完善不良贷款市场化处置机制，稳妥化解重点大户资金链、担保链风险，严厉打击恶意逃废债、非法集资等行为，深入开展互联网金融风险专项整治，实现不良贷款率“三连降”。

　　（二）发力供给侧，产业转型步伐加快。推进“三大板块”成形成势。全市工业投资增长28%，150个制造业龙头项目完成投资600亿元，安踏一体化、晶安光电三期、通达三期等建成或部分建成，中化乙烯、九牧智慧制造等加快推进，三安高端半导体、美的智慧家居等顺利开工，HDT高效太阳能电池、国脉生物科技等签约落地。传统、高新、重化产业产值比例为59∶16∶25。推进国家自主创新示范区建设。实施42项创新举措，新认定高新技术企业218家、科技小巨人领军企业132家。深入实施人才“港湾计划”，开展企业人才自主评价试点，修订高层次人才认定办法，新认定高层次人才1087人；入选国家“万人计划”、国务院特殊津贴各4人。推进制造业数控化智能化改造。推广应用数字化生产线127条、智能装备和工业机器人1330台（套），新增国家级智能制造项目3个、服务型制造示范项目4个，被授予国家级服务型制造示范城市。成立军民融合产业投资基金，新增“民参军”企业30家。推进“第三产业提升年”活动。服务业百大项目完成投资182亿元，新增服务业营收超百亿企业2家、限额以上商贸企业290家。完成快递业务量9.6亿件、增长30%，新增3A级以上物流企业14家，国际邮政互换局投入试运营。网商产业园新增入驻市场主体3.5万个。全市接待游客6613万人次、旅游收入1063亿元，分别增长20.8%、26%。推进特色现代农业提质增效。完成农林牧渔业总产值358亿元，划定水稻生产功能区59.8万亩。实施现代农业建设项目66个，完成年度投资15.9亿元。新增国家级农业龙头企业2家、“三品一标”认证46个，新建农业标准化示范基地300个，培育农业物联网示范企业19家。基本完成农村土地承包经营权的确权登记和国有农场改革。

　　（三）系统推改革，营商环境不断优化。推进41项国家级改革试点任务，落实打造一流营商环境的意见及细则。深化“放管服”改革。推进证照分离、相对集中行政许可权改革试点，实行个体工商户全程智能化登记。企业开办登记压缩至5个工作日内，全年市场主体增长57%，平均每天新增874户。新行政服务中心投用，92个单位进驻，医保、社保、燃气、水、电等1090个便民服务事项全部纳入；推行“一门一号一窗一网”改革，梳理发布“一趟不用跑”“最多跑一趟”清单；深入开展规范行政服务自由裁量权改革，形成市县对接的标准体系。推动民营经济高质量发展。制定支持民营企业健康发展行动方案及配套措施，出台加大信贷投放、无还本续贷管理、政策性融资担保机构绩效考核等举措，全市贷款余额增长5.3%；通过工业用地弹性出让、失业保险金支持企业稳岗等，降低企业用地用工成本；发布鼓励民营企业投资项目清单，支持民营企业未分配利润再投资，引导社会资本参与政府项目，累计落地PPP项目43个、总投资520.7亿元。推进国资国企改革。市级国资集团公司加快实体化、市场化运作，资产总额突破1300亿元。加快海丝先行区建设。对接外资项目262个、总投资267.5亿元，实际利用外资41.9亿元、增长9.4%，与海丝沿线国家和地区贸易额达911亿元，荣膺“一带一路”建设案例城市。获批国家级市场采购贸易方式试点。泉州晋江国际机场新增国际及地区航线5条，全年旅客吞吐量744.3万人次、增长39.4%；港口货物吞吐量1.3亿吨、集装箱239万标箱。举办联合国教科文总部“远方的故乡”阿拉伯文化展览、2018世界闽南文化节（迪拜）等对外文化交流活动24场，与俄罗斯迈科普市结为友好城市。出台促进泉台经济文化交流合作73条措施，金门供水工程顺利通水，泉金航线运送旅客突破16万人次。密切与港澳侨合作，“泉籍青年精英故乡行”成为品牌，海外重点侨社、侨领联络联谊得到拓展，侨务工作基础更加扎实。

　　（四）注重宜居度，城市面貌明显改善。环湾建成区面积达226平方公里，城镇化率达66.7%。加快古城提质。深入开展国家生态修复、城市修补试点，实施古城“七个一”示范工程；完成金鱼巷整治，开工小山丛竹公园、中山路保护提升示范工程，基本建成“东亚之窗”文创园、泉州美食城。常态化举办“润物无声”系列主题展览。推进新城集聚。全面启动东海后埔、台商区白沙、晋东新区等片区建设。福厦客专、兴泉铁路、厦漳泉城市联盟路、城东至北峰快速通道等加快推进，泉州火车站综合交通枢纽、市公共文化中心春节前可正式投用。开展高速铁路、高速公路沿线环境综合整治，37条城乡重要通道景观提升有序推进，水线9个公园陆续建成开放。加强城市治理。规范中心市区共享单车管理，开展内沟河、工地文明施工、渣土车文明行车、老旧小区等综合整治。实施浦西滞洪区二期等内涝治理项目18个，改造中心市区易积水点45处。执行市人大《关于加快全市垃圾分类处理利用的决议》取得初步成效，中心市区大件垃圾、园林垃圾初步实现“大分流”，泉州开发区、市直单位实现生活垃圾分类全覆盖，中心市区参与居民约2.4万户，各县（市、区）试点也扎实推进。开展国家“智慧汽车基础设施和机制建设试点”。实施“百日拆违行动”，全年拆除“两违”594万平方米。

　　（五）突出补短板，人民得到更多实惠。认真实施市委“四心”工程、民生“XIN”行动，顺利完成36件为民办实事项目。落实乡村振兴战略。组织实施19个专项行动方案。开展“千村整治、百村示范”美丽乡村建设131个；新增486个行政村建设1个以上生活污水深度处理设施，新改建三格化粪池8.1万户；所有乡镇建成生活垃圾转运系统，所有行政村实现生活垃圾常态化治理。改建农村路网355.3公里、公路安全生命防护工程530公里。全力打好脱贫攻坚战，农村建档立卡贫困人口实现稳定脱贫，152个“空壳村”全部消除，2436户贫困户住房条件得到改善，造福工程搬迁1443人。优先发展教育事业。新增公办幼儿园学位1.1万个，普惠性幼儿园覆盖率达75%。基本完成全面改薄五年规划，深入开展城区义务教育学校“小片区管理”、农村薄弱学校“委托管理”、集团化办学，推进教师“县管校聘”改革，新增中小学学位1万个。支持4所应用型本科高校和23所优质职业院校发展，福州大学晋江科教园投用。鲤城、丰泽获评教育强区。抓好关心下一代工作，强化青少年社会教育活动中心校外阵地作用。推进健康泉州建设。市妇产医院一期开诊，老年医院完工，全市新增医疗机构床位2550张。组建市第一医院医疗集团，在石狮、永春、德化试点建设县域紧密型医共体，7家三级医院建立现代医院管理制度。创建国家级基层卫生机构34家。开展医疗“暖心服务”三年行动，医疗服务总体满意度居全省前列。荣获全国基层中医药工作先进单位。新建基层老年体育活动中心10个。提升文化实力。“古泉州（刺桐）史迹”申遗取得阶段性成果。深入实施“万千百十”文化惠民服务，建成“百姓书房”73个、村（社区）综合性文化服务中心示范点24个，举办公益性专业剧团演出1332场，启动“文化泉州云”项目，获批创建国家公共文化服务体系示范区。新增国家级非物质文化遗产传承人15名，获评中国工艺美术之都。成功举办国际大体联亚洲杯、环泉州湾国际公路自行车赛。第十六届省运会收获金牌208枚。完善社会保障体系。新增城镇就业12.7万人。建立全市统一的城乡医疗救助制度，城乡居民基本医保住院实际报销比率提高9个百分点。实现城乡低保标准一体化，低保标准和补助水平进一步提高。城市、农村社区居家养老设施覆盖率分别达100%和57%，新增养老床位3840张。老龄、老干部工作得到加强，慈善事业稳步发展。制定实施促进房地产市场持续健康稳定发展八年规划，基本建成保障性安居工程1.5万套，安置房办证历史遗留问题有序解决，住房价格保持平稳。打好污染防治攻坚战。贯彻落实国家大气、水、土壤“十条”及省、市实施方案，认真执行市人大《关于进一步加强全市流域水环境保护的决议》。集中攻坚中央、省环保督察整改项目，石狮和晋江飞灰填埋场、城镇生活污水处理厂提标改造等一批重难点项目顺利完工。完成污染物排海总量控制、城市开发边界划定等13项改革任务。河湖长制工作经验获中央改革办总结推广，县级以上集中式饮用水水源地和主要流域水质达标率保持100%，建设安全生态水系49公里，基本建成流域环境生态样板工程7个，彭村水库建成投用。加大海漂垃圾综合治理，近岸海域优良水质面积比例达97.1%。深化环保设施与能力建设大会战，完成投资54.8亿元，市工业废物综合处置中心投入试运行。持续开展生猪养殖和农业面源污染整治。完成植树造林8万亩、水土流失治理40.7万亩、矿山生态修复228万平方米。永春成为国家生态文明建设示范县。维护社会安全稳定。扎实开展扫黑除恶专项斗争，推进电信网络犯罪综合整治，做好矛盾纠纷排查调处，信访违法行为集中整治经验被中央信访联席会议推广。加强食品药品监管，建设食品安全“一品一码”全过程追溯体系。完善安全生产风险分级管控和隐患排查治理双重预防机制，安全生产形势总体稳定。推动双拥工作迈上新台阶。健全军人优待政策，完善十项拥军举措，着力解决驻泉部队军改需求和军人“三后”问题。圆满完成“国际军事比赛—2018海上登陆赛”支前保障工作。民族宗教、统计、粮食、物价、方志、档案、人防、海防、气象、地震以及妇女儿童、青少年、残疾人、红十字会、工会等工作都取得新成效。

　　（六）坚持转作风，从严理政更为扎实。在政府系统兴起“大学习”热潮。落实中央八项规定及实施细则精神和省、市实施办法，弘扬“马上就办、真抓实干”优良作风，驰而不息纠正“四风”，坚决整治“为官不为”“庸懒散拖”等问题，狠抓巡视巡察发现问题整改。自觉接受市人大及其常委会监督，落实政府重大决策出台前向人大报告制度，主动配合市人大依法履职，认真办理人大代表建议444件。支持市政协履行职能，主动接受民主监督，加快协商成果转化落实，认真办理政协提案668件。扎实推进依法行政，稳妥实施政府机构改革。加大审计监督力度，政府廉政建设深入推进。

　　各位代表，过去一年的成绩来之不易，这是省委、省政府和市委正确领导的结果，是全市人民用勤劳、智慧、勇气拼出来的。在此，我谨代表市人民政府，向全市广大干部群众，人大代表、政协委员，各民主党派、工商联、无党派人士、各人民团体、离退休老同志，向驻泉省部属单位，驻泉部队、武警官兵，企业界人士和来泉创业务工人员，向广大港澳同胞、台湾同胞、海外侨胞、海内外朋友们，表示衷心的感谢和崇高的敬意！

　　我们对政府工作中的问题和不足有清醒的认识，主要是：经济运行稳中有变、变中有忧，外部环境复杂严峻，经济下行压力加大，外贸出口增幅由高走低，农林牧渔业总产值、第三产业增加值增幅预计无法完成年度目标；转型升级任重道远，新动能有待加快培育，百元GDP税收贡献低于全省平均水平，研发经费支出占GDP比重与年度目标还有较大差距，高层次、高技能人才集聚度不高；中心城市能级偏弱，与产业发展不相匹配，环湾同城化统筹力度有待加强；基础设施、教育、医疗、文化等基本公共服务存在短板；安全生产还有不少隐患，生态环境保护任务依然艰巨；少数政府工作人员服务意识不强、责任担当不够。对此，我们将增强忧患意识，不堕信心、不失希望，坚持问题导向，认真加以解决。

　　二、2019年工作目标任务

　　2019年是新中国成立70周年，是全面建成小康社会关键之年，是三年赶超承上启下重要一年。做好今年政府工作：必须以习近平新时代中国特色社会主义思想为指导，全面贯彻党的十九大和十九届二中、三中全会精神，坚持稳中求进工作总基调，坚持新发展理念，坚持高质量发展落实赶超，坚持以供给侧结构性改革为主线，坚持深化市场化改革、扩大高水平开放，继续打好三大攻坚战，进一步落实“六稳”工作，围绕市委“作答时代命题、聚力赶超攻坚”要求，呼应泉州人民新关切、新期盼，打好转型组合拳，建好城市展示面，造好活力大环境，努力开创“五个泉州”建设新局面，勇当“新福建”建设主力军。

　　今年经济社会发展主要预期目标是：全市生产总值增长8.5%-8.7%，农林牧渔业总产值增长2%-2.5%，工业增加值增长8%-8.5%，第三产业增加值增长10.1%-10.4%；一般公共预算总收入增长4.5%，一般公共预算收入增长3.5%；固定资产投资增长10%-13%；实际利用外资增长5%，出口增长2%；社会消费品零售总额增长11%，居民消费价格涨幅控制在3%左右；居民收入增长要实现和经济增长基本同步；完成节能减排降碳任务。

　　（一）着力赋能实体经济，推动高质量发展落实赶超

　　泉州是靠实体经济起家的，也要靠实体经济走向未来。必须大力挖掘传统产业新价值，发展新制造、新经济，加快新旧动能转换，建设“1234”现代产业体系。

　　坚定不移建设制造强市。紧抓传统、高新、重化“三大板块”，塑造泉州制造新形象。一是致力打造全国领先的民生消费品制造基地。立足纺织服装、鞋业、建材家居、食品饮料等千亿产业集群，注重利用技术创新和规模效应形成新的竞争优势。完善产业生态圈。实施龙头成长计划，支持增资扩营、建设总部、完善配套，抓好重大传统产业项目52个，认定产业龙头企业300家以上；大力发展标准代工、高端代工，引导龙头企业将技术标准、质量体系、管理模式向供应链上下游中小企业移植，培育省级以上制造业单项冠军10家、“专精特新”中小企业30家以上；突出在细分领域、重要工序开展“机器换工”，实施重点技改项目200个以上，推广数字化、智能化生产线100条以上，培育智能化标杆工厂、数字化车间等示范项目15个以上。争创全国质量强市示范城市。突破核心技术瓶颈制约。支持组建产业创新联盟，开展印染、后整理等关键材料和工艺的联合攻关，组织智能制鞋喷胶生产线、陶瓷阀芯组装机等重大专项研发，抢占价值链高端环节。实施消费品科技赋能计划。顺应“个性、定制、智能”消费趋势，引导企业植入现代科技和商业模式，既卖产品，又卖体验、卖服务。二是致力打造全国重要的半导体产业基地。全市域统筹布局产业链招商项目，围绕三安高端半导体、西人马等龙头，加快矽品、信达光电二期等配套项目投建步伐，培育壮大集成电路、化合物半导体、石墨烯等产业。三是致力打造全国一流的绿色石化基地。统筹泉港、泉惠园区发展，发挥联合石化、中化等“油头”带动效应，加快推进中化乙烯等23个石化产业重点项目建设，铸链发展石化新材料和精细化工，着力拉长“化尾”。

　　深入实施创新驱动战略。以国家自主创新示范区建设为抓手，集聚科技创新全要素，力争研发经费支出增长30%以上。突出企业主体地位，鼓励全市工业500强企业设立研发机构（团队）、开展研发活动；落实研发费用税前加计扣除政策，完善高新技术企业梯度培育和奖补机制，加强知识产权保护和运用，新增入库瞪羚企业40家，新培育高新技术企业140家以上。加快规划建设“泉州科技城”，一院一策引进“国字号”研发机构，支持中科院大学落地建设智能制造学院，提高产学研合作实效。创新推行科技特派员制度。认真落实人才“港湾计划”，完善以“用”为导向的人才评价机制，支持31家龙头企业自主认定高层次人才、享受同等政策待遇，引进产业发展急需的本科及以上人才1万人。

　　大力发展新产业新经济。紧抓“产业数字化”转型和“数字产业化”培育，抓好中国国际信息技术（福建）产业园、泉州软件园等重点载体建设，大力发展软件、集成电路设计、物联网、云计算、大数据等产业。推动人工智能与产业融合发展，争取在智能工业机器人、智能芯片等方面取得突破。加快发展工业互联网，抓好晋江“互联网+鞋业”、南安“互联网+泛家居”、德化“互联网+陶瓷”等省级链条化试点项目，打造3个以上行业领域工业互联网示范平台。培育壮大军民融合产业，支持企业承接“军转民”科技成果转化，参与军品研发生产，力争在传感器、卫星导航、新材料等领域实现突破。

　　加快现代服务业提质扩量。持续开展“第三产业提升年”活动。制造服务将立足生产基地优势，整合提升专业市场、物流园区，发展智慧物流、智慧仓储，培育第三方、第四方物流，确保3A级以上物流企业超百家；扶持建设创意孵化、工业设计、垂直电商、供应链管理、检验检测认证等示范平台，建设国家级服务型制造示范城市。消费服务将突出培育体验经济、全渠道经营、新零售等新业态，推动物业管理、家政服务行业规范化、标准化发展，新增限上商贸企业200家；落实好个人所得税专项附加扣除政策，增强消费能力，改善消费环境。文旅服务将按照“亮点在古城、厚度在山海、空间在生态连绵带”全域旅游发展思路，抓好“一社一游一点”三大提升工程，讲好泉州故事，发展六大旅游产品，培育“泉州伴手礼”品牌，力争接待游客、旅游收入均增长20%，打造海丝文化休闲旅游目的地。

　　做实高质量赶超的项目支撑。开展“项目攻坚2019”，进一步充实赶超项目库，加快“五个一批”项目梯度转化。实施在建重点项目460个、总投资7991亿元，力争开工项目75个、竣工75个。强化大招商、招大商“一把手”工程，完善“三个三”招商工作机制，充分发挥县域招商主体作用，探索在泉州开发区、台商投资区成立招商公司，突出以商招商、产业链招商和专业化招商，力促重大产业项目接续落地。

　　（二）着力推进乡村振兴，提升农业农村现代化水平

　　泉州全面建成小康社会，农村不能丢，农业要有特色，农民要有获得感。我们将注重质量，从容建设，推动乡村振兴战略落实落地。

　　建设现代农业。落实粮食安全行政首长责任制，加强粮食流通调控，推广“优质粮食工程”。推动传统农业转型升级，实施农业产业振兴建设项目工程包，启动农业龙头企业、农产品标准化基地、农产品初加工中心、“三品一标”认证农产品、特色产业村等“五百示范项目”，培育发展茶叶、水产、蔬果菌、花卉苗木等特色优势产业，建好安溪国家现代农业产业园。培育壮大乡村旅游业。完善水利基础设施，开工建设白濑水利枢纽主体工程，加快推进永春马跳水库、山美水库至惠女水库连通工程等项目，抓好清新流域、农村饮水、安全生态水系等民生水利项目。加强水土保持，深化重点区域水土流失综合治理。扎实做好防灾减灾工作。

　　建设美丽乡村。深化农村人居环境整治，扎实推进“一革命四行动”。继续实施农村厕所革命，新建乡镇公厕61座、农村公厕143座。实施农村垃圾治理行动，完善“户分类、村收集、镇中转、县处理”的城乡一体化垃圾处理模式，开展农村生活垃圾干湿分离试点。实施农村污水治理行动，新增467个行政村建设1个以上生活污水深度处理设施，新改建三格化粪池5.8万户，加强污水设施运营管理，持续提升污水处理率。实施农房整治行动，开展农房“平改坡”改造和裸房整治试点，有效解决农民违规建房、私搭乱建等问题。实施村容村貌提升行动，推进村庄“三边三清”，改造公共照明，整治杆线“蜘蛛网”；全面落实路长制，加快农村公路“单改双”，深入实施县、乡道安全生命防护工程，建设“四好农村路”。充分发挥农民主体作用，深化村民依法自治实践，建立村民评议干部制度，修订完善村民自治章程和村规民约，让村民自己“说事、议事、主事”。

　　建设殷实乡村。开展农民工创业联盟行动，加强新型职业农民培训，支持农民就地就近就业创业。推广扶贫“三百”工程，多渠道扶持壮大薄弱村集体经济。支持推动原中央苏区、革命老区、少数民族乡村加快发展。把保障稳定脱贫、提高脱贫质量放在首位，坚持“一个延伸”，将结对帮扶贫困户的时限及各项扶贫政策延伸至2020年，落实一个茶果菜园、一栏家禽家畜、一人实现就业“三个一”措施，做到生产扶持、建房补贴、医疗援助、就业引导、就学补助“五个到户”，有效防止返贫致贫。

　　（三）着力践行“晋江经验”，推动改革开放再出发

　　“晋江经验”是泉州历史荣誉的光环，更是未来前行的航标。我们将围绕重点领域和关键环节，大胆地试、勇敢地改，全力再创“晋江经验”新辉煌。

　　推动民营经济做大做强。帮民营企业就是帮自己人。始终坚持“两个毫不动摇”，弘扬企业家精神，发挥企业家作用。深入实施支持民营企业健康发展行动方案，加大对企业特别是中小企业的帮扶力度，把更大规模的减税降费、清理拖欠民企账款、灵活确定工业用地出让年限、处理产权历史遗留问题等工作，一件一件办好办实。落实党政领导与企业家恳谈会、涉企政策咨询委员会制度，完善“12345”企业服务热线，建立民营企业诉求受理处置反馈机制，实行“一对一”精准服务，打通政策落地“最后一公里”，构建“亲”“清”新型政商关系。

　　推动重大改革落细落地。深化营商环境提升行动，落实审批服务“马上办、网上办、就近办、一次办”，深化“材料瘦身”“减证便民”专项行动，全面推开“证照分离”改革，加强跨部门联合随机抽查综合监管；提升市行政服务中心“一站式”功能，加强县乡行政服务标准化建设，打造覆盖市、县、乡、村四级的一体化网上政务服务平台。深化金融服务实体经济改革，推动银行业机构落实小微企业贷款“两增两控”目标，提高政策性融资担保机构、产业投资基金、政府风险补偿基金等运作实效，推广实施“银税互动”，支持做大“无间贷”“助保贷”“科创贷”等产品规模，大力发展供应链金融，力争全市制造业贷款增速达到GDP增速水平；鼓励推动企业改制上市、登陆科创板，探索民营龙头企业实施市场化“债转股”，推进海峡股权泉州交易中心设立“股改板”；加大不良贷款处置力度，维护良好金融生态。全面加强预算绩效管理，处理好规范政府举债与保障赶超发展资金需求的关系，统筹使用预算建设资金，综合运用地方政府债券、PPP、国有资产运营等方式，提高资金使用效益。深化国资国企改革，加快实现从管企业向管资本转变，支持国有企业参股收购上市公司，建设运营小微工业园。创新产业园区管理机制，实行市场化、专业化运作，推动经营性用地、园区物业、基础配套设施资产化。深化农村集体产权、林权制度改革。完成第四次经济普查工作。

　　推动全面开放走深走实。扎实推进海丝先行区建设，形成更多可视性成果。开展“外资攻坚年”活动，推动100个重点外资合同项目到资落地，深化与2-3家台湾百大企业合作。实施外贸经营主体培育“双十、双百、双千”工程，办好海丝国际品牌博览会和泉州品牌“境外行”“海丝行”活动，支持企业全球化布局。用好石狮市场采购贸易方式试点，规划建设进口贸易促进创新示范区，发挥综合保税区、陆地港等平台优势，促进跨境电商、市场采购、保税加工融合发展，提升国际贸易便利化水平。稳妥推进泉州开发区扩区。提高台商投资区综合发展水平，力争在引进台资项目上取得重大突破；落实惠台惠企措施，加强对台民间交流交往，完善互联互通机制。密切与港澳侨双向交流合作，用好南洋华裔族群寻根谒祖综合服务平台，加强“四有”“三新”人士联络联谊，规划建设侨商总部，更好发挥侨资侨力侨智的作用。

　　推动区域发展协同协作。主动融入闽西南协同发展区，推进厦漳泉同城化建设。突出重大交通基础设施快捷联通，加快打造“协同区2小时、都市区1小时”交通圈。推动台商投资区、泉州“芯谷”与厦门园区对接，扩大思明园、湖里园、长汀园等合作实效。加强区域医疗卫生、教育、文体、旅游、环保等方面协同协作，促进共建共享。

　　（四）着力提升品位品质，建设现代环湾中心城市

　　泉州人对自己的城市爱之深、责之切。我们将开展“城市建设提速年”活动，外塑形象，内炼品质，加快提升综合承载力和宜居度，实现城产人融合发展。

　　发挥规划引领作用。启动新一轮城市总体规划修编，加强环湾新城核心区的整体谋划和重点塑造，抓好晋江下游两岸及入海口景观风貌、洛阳江流域整治等专项规划，开展重点片区城市设计。发挥新城建设统筹委员会作用，加强对重大规划、重大平台、重大设施和产业布局的总体把控。全面实施“多规合一”。稳妥推进行政区划调整。

　　优化城市功能布局。打造古城保护样板。深化国家生态修复、城市修补试点，抓好64个古城重点项目，全面推开“七个一”示范工程。科学布局文创产业、精品酒店、研学旅游、非遗展示等业态，持续办好古城徒步穿越活动。提高新城集聚效应。聚焦165平方公里新城陆域范围，实施106个新城重点项目，集中力量推进东海后埔、后渚、台商区白沙、晋东新区等片区开发改造，努力把“两江”入海口打造为城市形象高地。大力发展都市型产业，加快建设东海总部区，培育楼宇经济，完善环湾区域的居住、商务、酒店、文教、医疗等功能配套。高标准打造南北翼。加快泉州“芯谷”南安园区开发，推进泉港石化工业园安全控制区建设。立足城乡统筹，建好22个“镇级小城市”、13个特色小镇。

　　完善城市基础设施。建成城东至北峰快速通道，做好东海、金屿、百崎、武荣等跨江跨海通道前期，加快打通两重环湾、两重环城路网，完善环湾交通体系。推进兴泉铁路、福厦客专、厦漳泉城市联盟路等项目建设，抓好厦漳泉城际铁路R1线等项目前期，加快石湖、石井等口岸泊位建设，实施泉州晋江机场改扩建工程，完善泉州对外连接通道。加快给排水、供电、供气等老旧管网改造，全线开工海西天然气管网德化支线工程。加强住宅小区幼儿园、居家养老、社区服务等公益性配套用房的规划建设管理。

　　提升城市景观风貌。围绕打造山水田园城市，落实“一环一湾十廊道”规划，组织实施百崎湖、九十九溪等19个生态连绵带项目。抓好“市民常走、客人常走”道路景观建设，完成“两高”沿线整治，建成桃花山至观音山绿廊、大坪山步行环线、绿道二期工程，持续提升37条城乡重要通道的景观风貌。以城市摄影师视角，结合“四山两江”自然风光、标志性建筑，建设一批城市观景台。

　　加强城市精细管理。认真执行市人大《关于加快全市垃圾分类处理利用的决议》，完善分类收集转运处理链条，加快垃圾“大分流、小分类”，全面推进垃圾分类进校园。紧盯市民最期待的“关键小事”，抓好住宅加装电梯、老旧社区整治、公共交通、过街天桥、停车场、防洪排涝等项目，开展人行道净化和自行车专用道建设。加强城市公共安全及风险防范。出台规范线路敷设和用电行为指导意见，解决建筑表内电气火灾高发问题。深化城市执法体制改革，拓展“数字城管”平台功能，完成国家“智慧汽车基础设施和机制建设试点”。深化“两违”综合治理。

　　（五）着力传承历史文脉，打造泉州共同精神家园

　　千年海丝文化、闽南文化是泉州人骨子里的骄傲。我们将把握复兴城市文化的历史际遇，以文化人、以文塑城、以文惠民，让城市有根有魂、形神兼备。

　　提升城市文明程度。弘扬和践行社会主义核心价值观，深化“传家训、立家规、扬家风”“身边故事、共同铭记”等活动，推动全国文明城市创建常态化机制化。深化全国农村移风易俗工作试点，开展婚丧礼俗整治，持续推进“助力新泉州、建功新时代”志愿服务行动。构建联合惩戒工作机制，提高全社会诚信水平。

　　弘扬传承历史文化。坚持不懈推进“古泉州（刺桐）史迹”申遗，做好第八批国保申报工作。依法保护利用物质文化和非物质文化遗产，落实文物保护和安全“五纳入”，实现国保单位监测预警平台全覆盖。实施闽南文化保护示范工程，办好第四届海上丝绸之路国际艺术节，筹建海上丝绸之路博物馆，开工建设闽南教育基地，建成非物质文化遗产馆。编制实施历史文化名镇名村和传统村落保护规划，促进原有形态、生活方式延续传承，留住美丽乡愁。

　　完善公共文化服务。加快创建国家公共文化服务体系示范区，持续提升基层公共文化设施，建成投用歌舞剧院、“文化泉州云”项目，加快美术馆、“百姓书房”建设步伐，深化文艺精品创作工程。大力开展全民健身活动，认真筹备世界中学生运动会，办好国际大体联世界杯、2019环泉州湾国际公路自行车赛、第十一届市运会等赛事。推进国家体育产业联系点城市建设，培育发展竞赛表演、场馆运营、健身休闲等体育服务。

　　（六）着力守护绿水青山，加大生态文明建设力度

　　喝上干净的水，呼吸新鲜空气，头顶一片蓝天，是泉州老百姓的基本权利，更是政府的不懈追求。必须以最坚决的态度、最有力的举措，打赢污染防治攻坚战。

　　推进生态环境治理。实施大气减排治理项目40个，强化臭氧污染防控，抓好工业烟粉尘、道路和建筑施工扬尘、餐饮油烟等污染源整治，建成机动车尾气遥感监测系统。认真执行市人大《关于进一步加强全市流域水环境保护的决议》，严格落实河湖长制，强化以流域为单元的系统治理，实现小流域Ⅲ类以上水质比例达86%以上。加大土壤污染管控和修复力度，实行危废信息化全过程监管。深化环保设施与能力建设攻坚，加快推进垃圾焚烧设施和餐厨垃圾、建筑垃圾处理设施建设。加强应急物资储备，提升环境应急能力，守住环境安全底线。

　　推进生态文明试验。完善制造业转型升级与生态文明建设正向互动机制，实施绿色制造工程，推动绿色产品、绿色工厂、绿色园区和绿色供应链发展。落实生态环境保护工作职责，坚决抓好环保督察、海洋督察反馈问题整改。完成综合性生态保护补偿试点、自然资源产权制度等7项改革任务。

　　推进节约循环发展。落实环保倒逼机制，加快淘汰落后工艺和过剩产能；增强服务意识，帮助企业制定环境治理解决方案。开展重点企业能耗、水耗、物耗、污染物排放对标改造行动，建好德化陶瓷、泉港石化等国家级循环经济园区。实施森林质量提升工程。有效盘活闲置厂房，依法清理闲置土地。发展钢结构等装配式建筑，推动建筑业转型升级。

　　（七）着力增进民生福祉，不断实现人民对美好生活的向往

　　市委“四心”工程、民生“XIN”行动和为民办实事项目深得人心，我们必须高标准扎实抓好，逐步把泉州人民的烦心事一件一件解决好，让人民的生活随着经济发展一年比一年好。

　　办好人民满意的教育。新增公办幼儿园学位1.3万个，确保普惠性幼儿园覆盖率达80%。持续推动义务教育优质均衡发展，创建一批义务教育管理标准化学校。支持乡村小规模学校和乡镇寄宿制学校建设。深入实施消除义务教育阶段大班额专项规划。开展小学课后服务试点。推进“双一流”、应用型本科高校和省示范性现代职业院校建设，抓好首批23个产教融合实训基地，争创国家产教融合型试点城市。

　　统筹实施教育“领航团队”培养工程、乡村教师素质提升工程，落实提高乡村教师生活补助政策，探索推进编外合同教师择优入编。抓好老年教育，办好继续教育、特殊教育。加强未成年人思想道德建设和心理健康服务，精准关爱下一代。

　　建设公平普惠的健康泉州。加快发展、合理布局优质医疗卫生资源，抓好市一院二期、医高专附属人民医院、妇产医院二期、颐和医院等14个重点医疗项目，支持华侨大学建设附属医院，鼓励支持社会办医，全市新增床位3500张。深化医改工作，县级以上二级公立医院实行院长年薪制和目标考核制，三级医院全面建立现代医院管理制度，深入推进市第一医院医疗集团和石狮、永春、德化县域紧密型医共体建设。实施高端人才和实用型人才培养工程，重点培养引进儿科、产科、重症医学、精神卫生等紧缺薄弱学科的人才，加强全科医生队伍建设。提高家庭医生签约服务质量。推进乡村卫生服务一体化管理。深入开展“暖心服务”行动，进一步加强医风医德建设。抓好公共卫生服务管理，提升疾病防控能力，创建一批老年人健身康乐家园。完善基层妇幼健康体系，服务好全面两孩政策。发挥好计生协会作用。健全食品药品安全风险隐患排查制度，巩固深化食品药品网格化监管，毫不松懈抓好非洲猪瘟防控，争创国家食品安全示范城市。

　　完善多层次的社会保障体系。实施就业优先政策，支持企业稳定岗位，促进高校毕业生、退役军人、就业困难人员、农民工更高质量更充分就业创业。继续实施全民参保计划，完善大病医保制度，优化医疗救助政策。优先发展居家社区养老服务，支持12个街道和重点乡镇建设照料中心、139个村建设养老服务站，推广智慧养老服务。加快推进医养结合，力争护理型养老床位达40%，养老机构医养结合率100%。培育孵化专业化服务组织，支持社会力量兴办养老机构。持续开展“救急难”工作。坚持房子是用来住的、不是用来炒的定位，完善住房市场体系和住房保障体系，持续推进棚户区、农村危房、石结构房屋改造，建立稳定房价长效机制。认真实施妇女儿童发展纲要，关爱留守老人、留守儿童，发展好残疾人事业、社会福利和慈善事业。

　　构建现代化治理体系。夯实公安基层基础，推进“城安系统”建设，纵深开展扫黑除恶专项斗争，加强电信网络犯罪综合整治，抓好信访法治化建设，打造更高水平“平安泉州”。落实地方党政领导干部安全生产责任制及省、市实施细则，坚持安全生产“四个一定”，突出隐患零容忍、预案实战化、处置专业化，营造安全发展环境。支持工会、共青团、妇联等人民团体发挥更大作用。深入开展民族团结进步创建活动，落实宗教工作三级网络、两级责任制。深化双拥共建，服务驻泉部队深化国防和军队改革，抓好民兵综合训练基地项目，维护军人军属和退役军人合法权益，落实军人荣誉体系建设，巩固发展军政军民团结良好局面。

　　三、加强政府自身建设

　　各位代表！新的一年，我们承载着更大期盼，肩负着更大责任。政府全体工作人员将全面增强“八种本领”，不断提升履职能力，努力建设让人民满意的新时代服务型政府。

　　提高政治站位，更自觉更坚定。把政治建设放在政府自身建设的首位，把党的领导贯穿政府工作全过程各方面，树牢“四个意识”，坚定“四个自信”，坚决做到“两个维护”。贯彻执行中央方针政策，认真落实省委、省政府和市委决策部署。以最坚决的态度、最严格的要求，抓好中央、省委巡视反馈问题的整改。落实意识形态工作责任制。

　　强化责任担当，马上干务实干。围绕高质量发展落实赶超，敢于担当，善于作为，细化、量化、实化政府工作，层层传导压力、层层压实责任。大力倡导“马上就办、真抓实干”，建立主要任务工作台账，实行清单化、项目化管理，做到一个专项、一套方案、一抓到底，全面提升行政效能。坚持激励和严管并举，注重在一线繁重岗位锤炼政府干部队伍，营造干事创业浓厚氛围。

　　严格依法行政，讲规矩讲法治。开展“法治政府建设年”活动，实施“七五”普法规划，提升宪法意识，坚持用法治思维和法治方式深化改革、推动发展、化解矛盾、维护稳定。落实政府机构改革，优化职能配置。加快政府规章制定，推行行政执法三项制度，推进政务公开，完善政府法律顾问制度。严格执行市人大及其常委会决议决定，自觉接受市人大法律监督和工作监督，主动接受市政协民主监督，高质量办理建议提案。认真听取各民主党派、工商联、无党派人士和人民团体意见建议。深化府院委联动互动。

　　加强廉洁自律，筑防线守底线。坚定不移推进政府系统党风廉政建设，加大正风肃纪、反腐惩恶力度。严格落实中央八项规定及实施细则精神和省、市实施办法，持之以恒纠正形式主义、官僚主义新表现。凡是群众反映强烈的问题都严肃认真对待，凡是损害群众利益的行为都坚决纠正，以政府的“清廉指数”提升人民的“满意指数”。

　　各位代表！在高质量赶超征程中，每一位泉州人都是主角，每一个政府工作人员都有责任。让我们更加紧密地团结在以习近平同志为核心的党中央周围，在省委、省政府和市委的坚强领导下，提振爱拼敢赢的精气神，与全市人民一起拼搏、一起奋斗，以优异的成绩迎接新中国成立70周年！
